# Exosomal circEIF3K from cancer-associated fibroblast promotes colorectal cancer (CRC) progression via miR-214/PD-L1 axis

**DOI:** 10.1186/s12885-021-08669-9

**Published:** 2021-08-19

**Authors:** Kaihua Yang, Jie Zhang, Chuanqing Bao

**Affiliations:** 1 Department of Radiotherapy, Affiliated Hospital of Jiangnan University, Wuxi, China; 2 Medical School, Nantong University, Nantong, China; 3 Department of Gastrointestinal Surgery, Affiliated Hospital of Jiangnan University, No. 1000 Hefeng Road, Wuxi, 214122 China

**Keywords:** Colorectal cancer, Tumorigenesis, cancer-associated fibroblast, Exosome, circEIF3K, miR-214

## Abstract

**Background:**

Tumor microenvironment (e.g., cancer-associated fibroblast) plays a key role in cancer tumorigenesis and metastasis. However, the detailed mechanism of whether hypoxia promotes CRC progression via tumor microenvironment remains unclear.

**Methods:**

In this study, circEIF3K exosome was examined by NanoSight Tracking Analysis and RT-qPCR. We used cell colony formation assay, transwell assay and tube formation assay to determine proliferation, invasion and metastasis of HCT116 or SW620 cells. Xenograft tumor assay was employed to show in vivo tumor growth of HCT116 cells.

**Results:**

We found that hypoxia could induce secretion of circEIF3K exosome. Conditioned medium (CM) and exosome from circEIF3K knockdown CAF significantly attenuated proliferation, invasion and tube formation of HCT116 or SW620 cells, which could be reverted by miR-214 under hypoxia treatment. Besides, we observed that circEIF3K knockdown evidently impaired tumor growth in mice. TCGA dataset analysis showed that low expression of circEIF3K was observed in normal tissues and associated with prolonged survival time. Finally, PD-L1 was confirmed as important target for miR-214 in CRC.

**Conclusion:**

In conclusion, our study reveals that a novel axis circEIF3K/miR-214/PD-L1 mediates hypoxia-induced CRC progression via CAF, providing the rationale for developing new targeted therapeutics to treat CRC.

## Background

Colorectal cancer is the third common tumor type and caused cancer-related mortality [1]. 5-year survival rate is 65% [2]. In general, most of CRC tumor will progress to liver metastasis state [3]. Tumor microenvironment plays a critical role in cancer progression. Of which, hypoxia is a common feature of solid tumors, which contributes to tumorigenesis and metastasis [4]. Recently, some studies reported that hypoxia regulated cancer progression via promoting secretion of circRNAs exosome [5, 6].

circRNA are originally regarded as non-functional junk RNA. However, some recent evidences showed that circRNAs are involved in a variety of pathological processes, such as tumors [7, 8]. In CRC, some circRNAs are found to regulate tumor phenotypes. CircCCDC66 regulated miR-3140/autophagy to promote CRC tumorigenesis [5]. Has_circ_0000826 was associated with CRC metastasis [6]. To date, circEIF3K was shown to modulate apoptosis and autophagy in bacterial cells [9]. Unfortunately, whether circEIF3K can affect cancer biology remains unknown.

miRNAs are usually 18-22 nt non-coding RNAs [10]. They regulate gene expression levels via binding 3′-UTR of mRNAs, which leads to aberrant expression of target genes [11]. A number of studies showed that miRNAs were dysregulated in various types of cancer cells [12, 13]. MiR-214 has been demonstrated to inhibit HCC [14], bladder cancer [15] and CRC [16] progression. Thus, we hypothesized that miR-214 might be potential target of circEIF3K in CRC. Programmed death ligand-1 (PD-L1, also known as CD274) is a negative immune regulator via blockage of T cell activation signaling [17]. Interestingly, PD-L1 is overexpressed in many tumors. Sun et al. revealed that miR-214 targeted PD-L1 in diffuse large B-cell lymphoma [18]. Based on these findings, we hypothesized that PD-L1 might act as downstream effector for circEIF3K in CRC.

In this study, the purpose is to illuminate the underlying mechanism of circEIF3K-regulated CRC tumorigenesis and metastasis. We attempted to investigate the role of miR-214/PD-L1 axis in CRC. The conclusions will advance our understanding of CRC pathogenesis and treatments.

## Materials and methods

### Cell culture and treatment

Human colorectal cancer cells HCT116 and SW620, and human embryonic kidney cell 293 T were all purchased from Shanghai cell bank of Chinese Academy of Sciences. Normal colon epithelial cell FHC were from ATCC. All cells were cultured in Dulbecco’s Modified Eagle’s Medium supplemented with Fetal Bovine Serum (FBS, 10%). Human dermal lymphatic endothelial cells (HDLEC) (PromoCell) were cultured in endothelial cell growth medium at 5% CO_2_, 37 °C. For hypoxic treatment, the cells were cultured under hypoxia (94% N_2_, 5% CO_2_, 1% O_2_) or normoxia (20% O_2_, 5% CO_2_). 2 mg/ml Actinomycin D (Shanghai Haoran) was used to treat HCT116 cells for 24 h.

### Generation of stable cell lines

sh-NC, sh-circEIF3K-1 and sh-circEIF3K-2 were introduced along with lentivirus packaging vectors (pVSVG and pPAX2) into 293 T cells by Lipofectamine 2000 (invitrogen). The viruses-containing media were collected after 48 h of transfection. The viruses were then used to infect CRC cells. To obtain stable cells, 2μg/ml puromycin was used to treat the cells for about 7 days. shNC denotes scramble vector.

- sh-NC: UACCUUGAAGCCUUAAAACCU
- sh-circEIF3K-1: GACUCAGCCGUACCAGUUCAA
- sh-circEIF3K-2: GUUGACUCAGCCGUACCAGUU

### Isolation of cancer-associated fibroblasts

CAFs were isolated according to previously described [19]. The cells were immortalized by transfecting with human telomerase reverse transcriptase. The medium of CAFs were termed as conditioned medium (CM). In this study, CM of CAFs treated with mock or hypoxia were named as mock-CM or hypoxia-CM. This study was approved by ethics committee of affiliated hospital of Jiangnan university, and informed consents were acquired from the patients before this study.

### Cell transfection

We utilized Lipofectamine 2000 (invitrogen) to transfect miR-214 mimic or inhibitor (~ 50 uM) into HCT116 or SW620 cells. The cells were collected and used for subsequent analysis after 48 h of transfection.

### Colony formation assay

CRC cells (2 × 10^4^ cells) were seeded in 6-well plate and cultured for 2 weeks. Then, the cell colonies were fixed with 4% paraformaldehyde reagent. Next, 0.005% crystal violet was used to stain the colonies. For CM treatment, the old CM was replaced with fresh CM every 3 days.

### Tube formation assay

In this experiment, 24-well plates were coated with Growth Factor Reduced (GFR) matrigel at 37 °C for 2–3 h. We seeded HDLEC cells (~ 20,000 cells/well) into coated plates. Formed tubes were observed and captured by microscopy (× 200 magnification). CM treatment was performed during this experiment.

### Invasion assay

HCT116 or SW620 cells (1 × 10^5^ cells) were suspended in 200 μL of media without FBS. Top chamber contains 8 μm pore membrane. Meanwhile, media with 10% FBS was added to the bottom chamber. After 24 h, invaded cells were stained with crystal violet. The invaded cell number was counted for statistical analysis.

### RT-qPCR and RNase R treatment

Trizol (invitrogen) was used to lyse the cells, and RNAs were dissolved in RNase-free ddH_2_O. To differentiate circRNA and mRNA, RNAs were treated with RNase R (Epicenter) at 37 °C for 2–3 h. 1 μg of total RNAs were reverse transcribed using PrimeScript Kit (TAKARA). Real-time PCR was carried out using SYBR Green reagent. Thermocycling conditions were used: Initial denaturation at 95 °C for 5 min, followed by 40 cycles of 95 °C for 30 s, 60 °C for 30 s and 72 °C for 30 s.

- circEIF3K-F: TGACAGTGTGTCCAGCATCA
- circEIF3K-R: AAGTCTGTGTGCGGCAAGTT
- miR-214-F: TCTGCCTGTCTACACTTGCT
- miR-214-R: TGTACAGGTGAGCGGATGTT
- PD-L1-F: TGCCACCCACTGTCCTTTTA
- PD-L1-R: GTTTTCCCCTCGCATCATCC
- Actin-F: TGGCATCCACGAAACTACCT
- Actin-R: TCTCCTTCTGCATCCTGTCG

### Isolation of exosome

CAFs were seeded overnight, supernatants of media were collected and centrifuged at 10,000×g for 30 min to discard cell debris. Then, the supernatants were centrifuged at 120,000×g for 2 h to collect vesicles.

### NanoSight tracking analysis

The CAFs were seeded in 12-well plate and cultured in DMEM for 24–36 h. Then, the supernatant were collected and centrifuged at 120,000×g for 30 min, 4 °C.Supernatants and exosomes were analyzed on NanoSight LM10.

### Xenograft

We performed this experiment using 6 immunodecifient male NOD-SCID mice (3 mice per group). Mice were injected subcutaneously with HCT116 cells (2 × 10^6^). Cells resuspended in 100 μL of PBS were injected into the tail vein. We measured tumor weight and volume. Tumor volume = 0.5 × length × width^2^. This study was approved by ethics committee of affiliated hospital of Jiangnan university.

### TCGA

Datasets of CRC patients were downloaded from TCGA (275 tumors and 349 normal tissues). The circEIF3K expression levels were shown in normal and tumor tissues. In addition, overall survival and stage plot of CRC patients were also shown.

### Statistical analysis

All data in this study were presented as mean ± SD. Chi-squared test, Student’s t test, One-way ANOVA were performed for comparisons. ^*^P < 0.05 was statistically significant.

## Results

### Hypoxia stimulated exosomal circEIF3K secretion from CAF

**Fig. 1:**
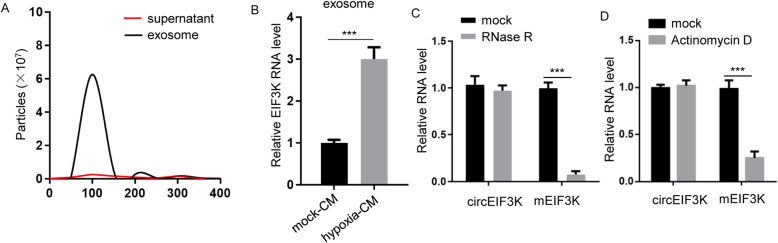
Hypoxia stimulated exosomal circEIF3K secretion from CAF. **A** NanoSight quantification of solution or exosome from conditioned media of CAFs under hypoxia treatment. **B** RT-qPCR showed circEIF3K level in exosome fraction of mock-CM or hypoxia-CM of CAFs. ***p < 0.001. **C** RT-qPCR showed RNA level of circular RNA (circEIF3K) or mRNA (mEIF3K) treated with or without RNase R. ***p < 0.001. **D** RT-qPCR showed RNA level of circular RNA (circEIF3K) or mRNA (mEIF3K) in HCT116 cells treated with or without Actinomycin D. ***p < 0.001

Hypoxia has been shown to enhance cancer progression; however, the detailed mechanism remains unclear. We hypothesized that hypoxia might contribute to cancer progression via tumor microenvironment (e.g., cancer-associated fibroblast). To validate, the CAFs were cultured and treated with normoxia or hypoxia. We found that CAFs could secrete exosomes under hypoxia treatment (Fig. 1A). More interestingly, we also observed that circEIF3K was existed in exosome. The level of exosomal circEIF3K in CAF media treated with hypoxia was higher (Fig. 1B). To confirm whether the exosomal RNA was circRNA, we used RNase R to treat circEIF3K. EIF3K mRNA (mEIF3K) was used as positive control (Fig. 1C). In addition, we used Actinomycin D to treat HCT116 cells and measured circEIF3K and EIF3K mRNA (Fig. 1D). The result showed that RNase R and Actinomycin D could decrease EIF3K mRNA, not circEIF3K.

### circEIF3K depletion in CAF led to attenuated CRC progression

**Fig. 2:**
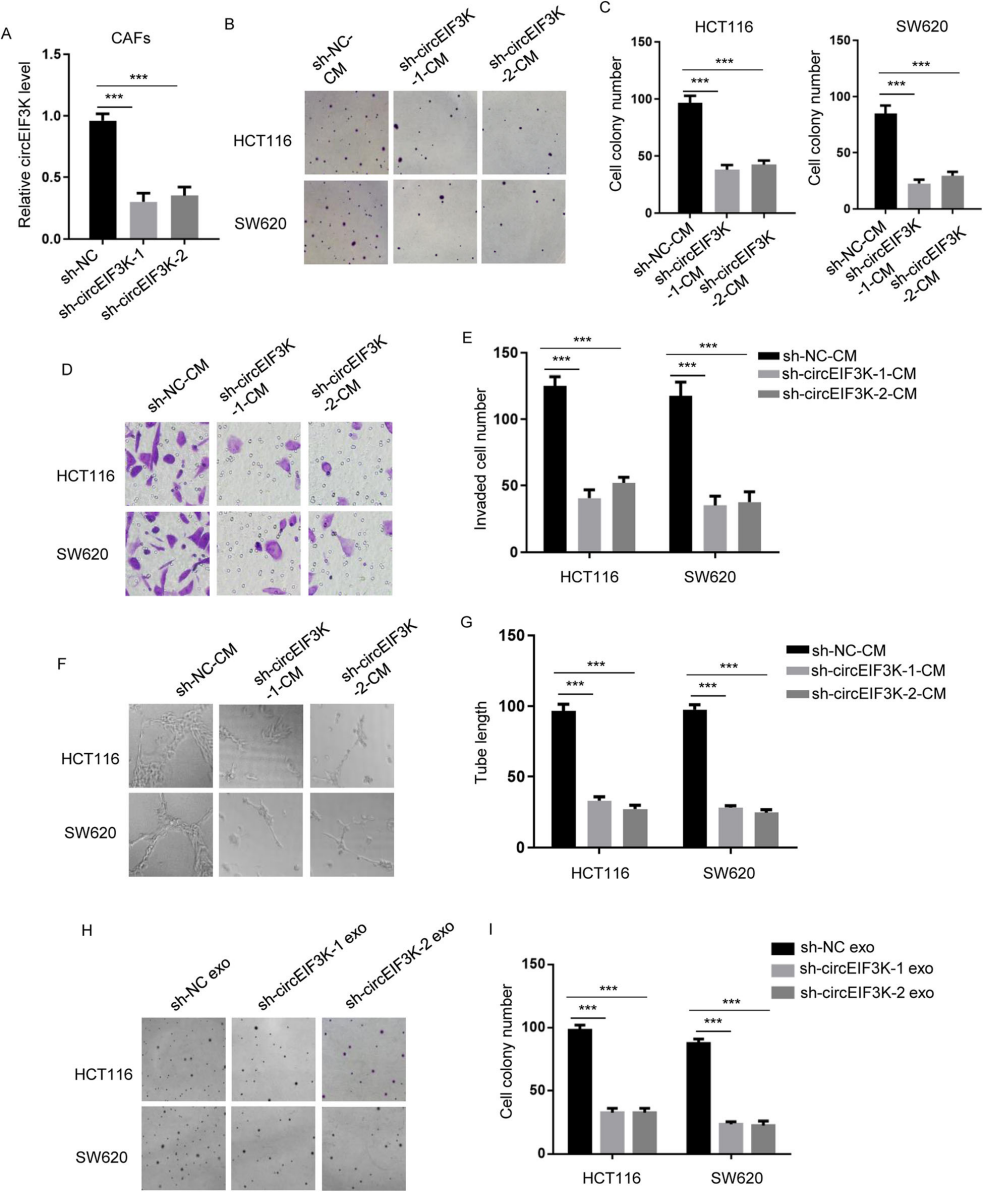
circEIF3K depletion in CAF led to attenuated CRC progression. **A** circEIF3K expression levels were shown in CAFs stably transfected with sh-NC, sh-circEIF3K-1, sh-circEIF3K-2 by RT-qPCR. ***p < 0.001. **B** and **C** Cell proliferation of HCT116 or SW620 cells incubated with CM of CAFs stably transfected with sh-NC, sh-circEIF3K-1, sh-circEIF3K-2 by cell colony formation assay under hypoxia. ***p < 0.001. **D** and **E** The invasion of HCT116 or SW620 cells incubated with CM of CAFs stably transfected with sh-NC, sh-circEIF3K-1, sh-circEIF3K-2 were determined by transwell assay under hypoxia. ***p < 0.001. **F** and **G** Tube lengths of HDLEC cells (sh-NC-CM, sh-circEIF3K-1-CM, sh-circEIF3K-2-CM) were measured by tube formation assay under hypoxia. ***p < 0.001. **H** and **I** Cell proliferation of HCT116 or SW620 cells incubated with exosomes derived from CAFs stably transfected with sh-NC, sh-circEIF3K-1, sh-circEIF3K-2 by cell colony formation assay under hypoxia. ***p < 0.001

As circEIF3K could be released by CAF under hypoxia, we attempted to investigate the effect of circEIF3K knockdown in CAF on CRC cell tumorigenesis. RT-qPCR showed that circEIF3K was markedly decreased in sh-circEIF3K-1 and sh-circEIF3K-2 cells (Fig. 2A). Then, we performed cell colony formation assay to examine proliferation of HCT116 or SW620 cells incubated with media of CAF. circEIF3K knockdown significantly led to decrease in cell colony number (Fig. 2B and C). Transwell assay results demonstrated that CRC cells incubated with media of sh-circEIF3K CAF exhibited less invaded cells (Fig. 2D and E). Likewise, tube formation assay showed that tube length of HDLEC was attenuated by media of CRC cells preincubated with CM of sh-circEIF3K CAF (Fig. 2F and G). Eventually, we treated HCT116 or SW620 cells with exosomes derived from sh-NC or sh-circEIF3K CAFs. The results demonstrated that CRC cells treated with exosomes from sh-circEIF3K CAFs exhibited less cell colony number than CRC cells treated with exosomes from sh-NC CAFs (Fig. 2H and I). In conclusion, our data suggested that circEIF3K depletion in CAF inhibited CRC progression.

### circEIF3K modulated CRC progression in animal and patients

**Fig. 3:**
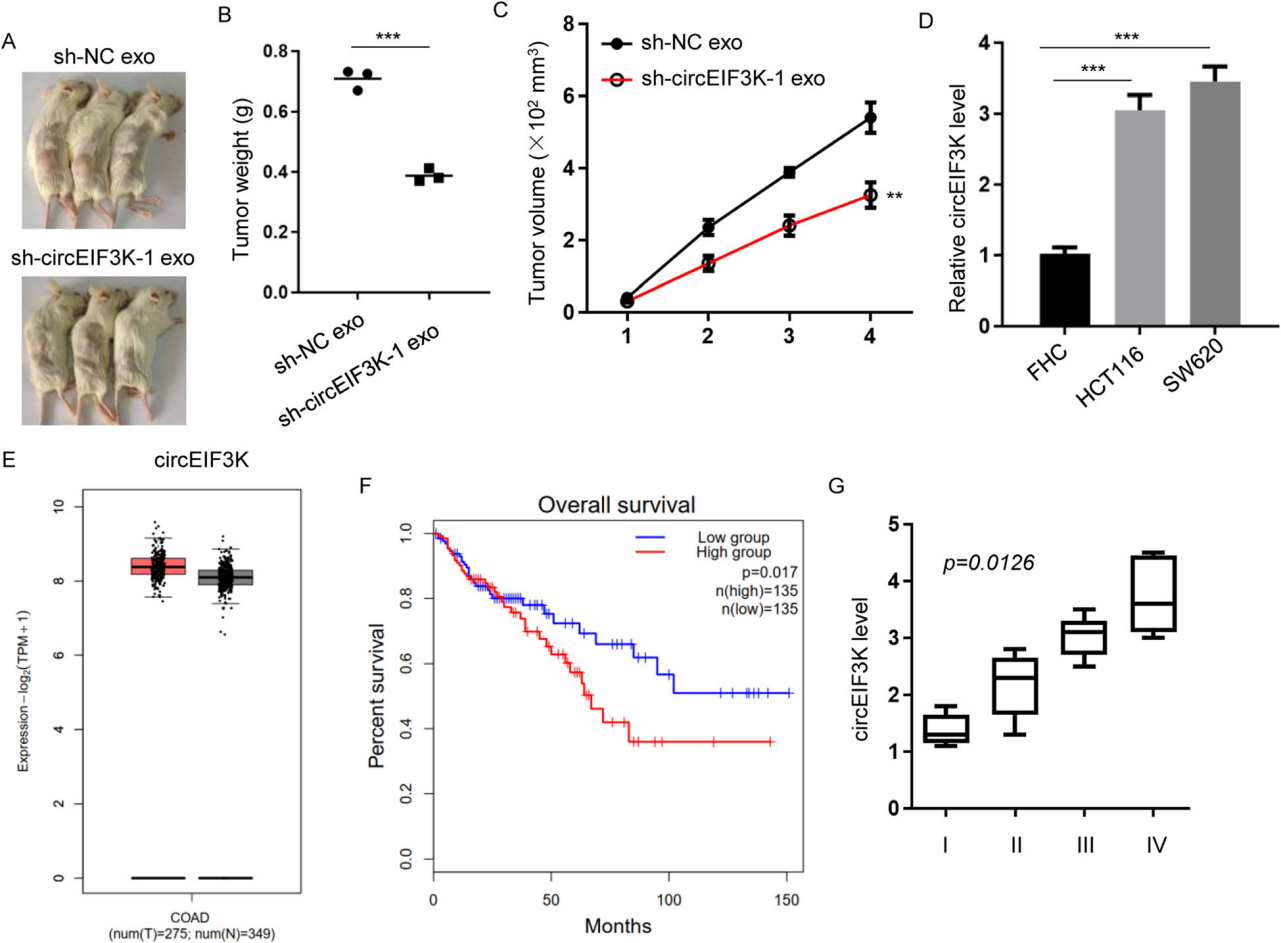
circEIF3K modulated CRC progression in animal and patients. **A** In vivo tumor growth of HCT116 cells treated with sh-NC exosome (sh-NC exo), sh-circEIF3K-1 exosome (sh-circEIF3K-1 exo) were examined in NOD-SCID mice (*n* = 3). **B** and **C** Tumor weights (**B**) and volumes (**C**) in Figure 3A were measured. **p < 0.01; ***p < 0.001. **D** RT-qPCR showed circEIF3K levels of normal colon epithelial cell FHC and CRC cell lines (HCT116 and SW620 cells). ***p < 0.001. **E** TCGA dataset analysis showed circEIF3K levels of CRC tumors (*n* = 275) and adjacent normal tissues (*n* = 349). **F** Overall survival of CRC patients with low circEIF3K level group (*n* = 135) and high circEIF3K level group (*n* = 135). *p* = 0.017. **G** Stage plot of CRC patients showed association of circEIF3K levels with stages. *p* = 0.0126 (stage IV vs stage I)

In order to investigate the role of circEIF3K in xenograft tumor growth, HCT116 cells were utilized to inoculate subcutaneously into NOD-SCID mice (Fig. 3A). Then, the mice were injected with exosomes from sh-NC or sh-circEIF3K CAFs. Tumor volume and weight data revealed that sh-NC exosome-treated tumors were larger than sh-circEIF3K exosome (Fig. 3B and C).

In addition, we evaluated circEIF3K levels in normal colon epithelial cell FHC and two CRC cell lines (HCT116 and SW620) by RT-qPCR. We found that circEIF3K was elevated in CRC cell lines compared to FHC (Fig. 3D). We analyzed circEIF3K levels in normal tissues and CRC tumors in TCGA datasets. Consistent with data in cell lines, we also observed increase in circEIF3K levels in CRC tumor patients (Fig. 3E). Patients with low level of circEIF3K exhibited prolonged survival rate in relative to high group (Fig. 3F). Moreover, expression levels of circEIF3K was higher in advanced stages (III + IV) in relative to low stages (I + II) (Fig. 3G). Together, circEIF3K exerted oncogenic role in animal and clinics.

### miR-214 was key for hypoxia-induced tumorigenesis of CRC

**Fig. 4:**
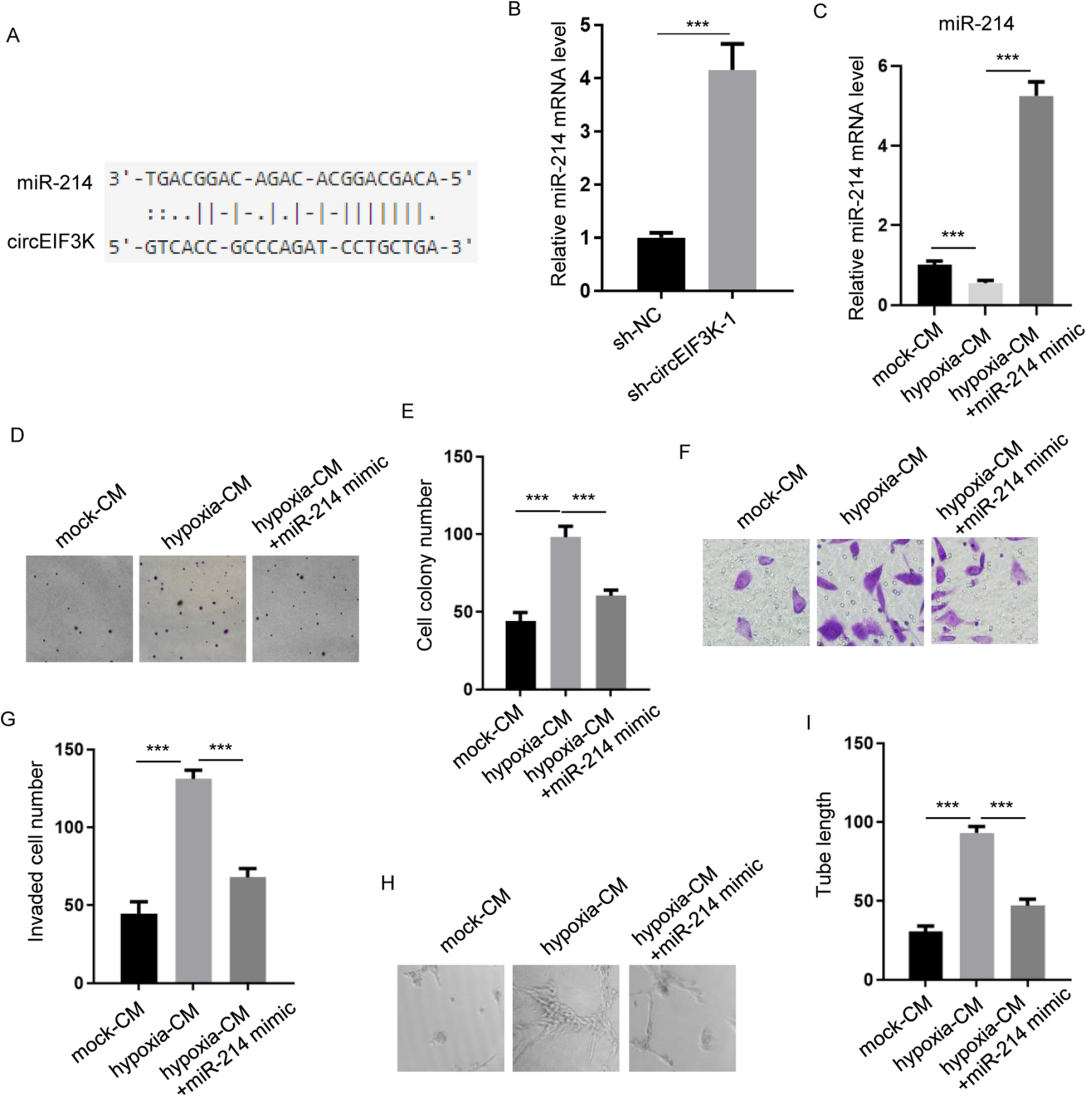
miR-214 was key for hypoxia-induced tumorigenesis of CRC. **A** StarBase 2.0 predicted the interaction between circEIF3K and miR-214. **B** miR-214 levels were examined by RT-qPCR in HCT116 cells stably transfected with sh-NC or sh-circEIF3K-1. ***p < 0.001. **C** miR-214 expression levels were detected by RT-qPCR in HCT116 cells treated with mock-CM, hypoxia-CM, hypoxia-CM + miR-214 mimic. ***p < 0.001. **D** and **E** Cell proliferation of HCT116 cells treated with mock-CM, hypoxia-CM, hypoxia-CM + miR-214 mimic were examined by cell colony formation assay. ***p < 0.001. **F** and **G** Invasion of HCT116 cells treated with mock-CM, hypoxia-CM, hypoxia-CM + miR-214 mimic were measured by transwell assay. ***p < 0.001. **H** and **I** Tube lengths of HDLEC cells (mock-CM, hypoxia-CM, hypoxia-CM + miR-214 mimic) were measured by tube formation assay. ***p < 0.001

To figure out the potential molecular mechanism of circEIF3K-promoted CRC, online predict program StarBase v2.0 was utilized to identify miR-214 as one of the downstream effectors (Fig. 4A). RT-qPCR data showed that circEIF3K knockdown increased miR-214 level in HCT116 cells (Fig. 4B). Because hypoxia could induce secretion of exosomal circEIF3K from CAF, we sought to explore whether miR-214 could rescue hypoxia-mediated phenotypes. We first examined miR-214 levels in HCT116 cells treated with normoxia, hypoxia or hypoxia plus miR-214 (Fig. 4C). Expectedly, we found that hypoxia evidently increased cell colony number, while miR-214 mimic reverted hypoxia-enhanced cell colony formation ability (Fig. 4D and E). Besides, transwell assay demonstrated that hypoxia induced invasion of CRC cells, which could be attenuated by miR-214 mimic (Fig. 4F and G). Finally, tube formation assay revealed that hypoxia substantially stimulated tube formation of HDLEC, whereas this enhancement was largely restored by miR-214 (Fig. 4H and I).

### miR-214 downregulated PD-L1 expression in CRC

**Fig. 5:**
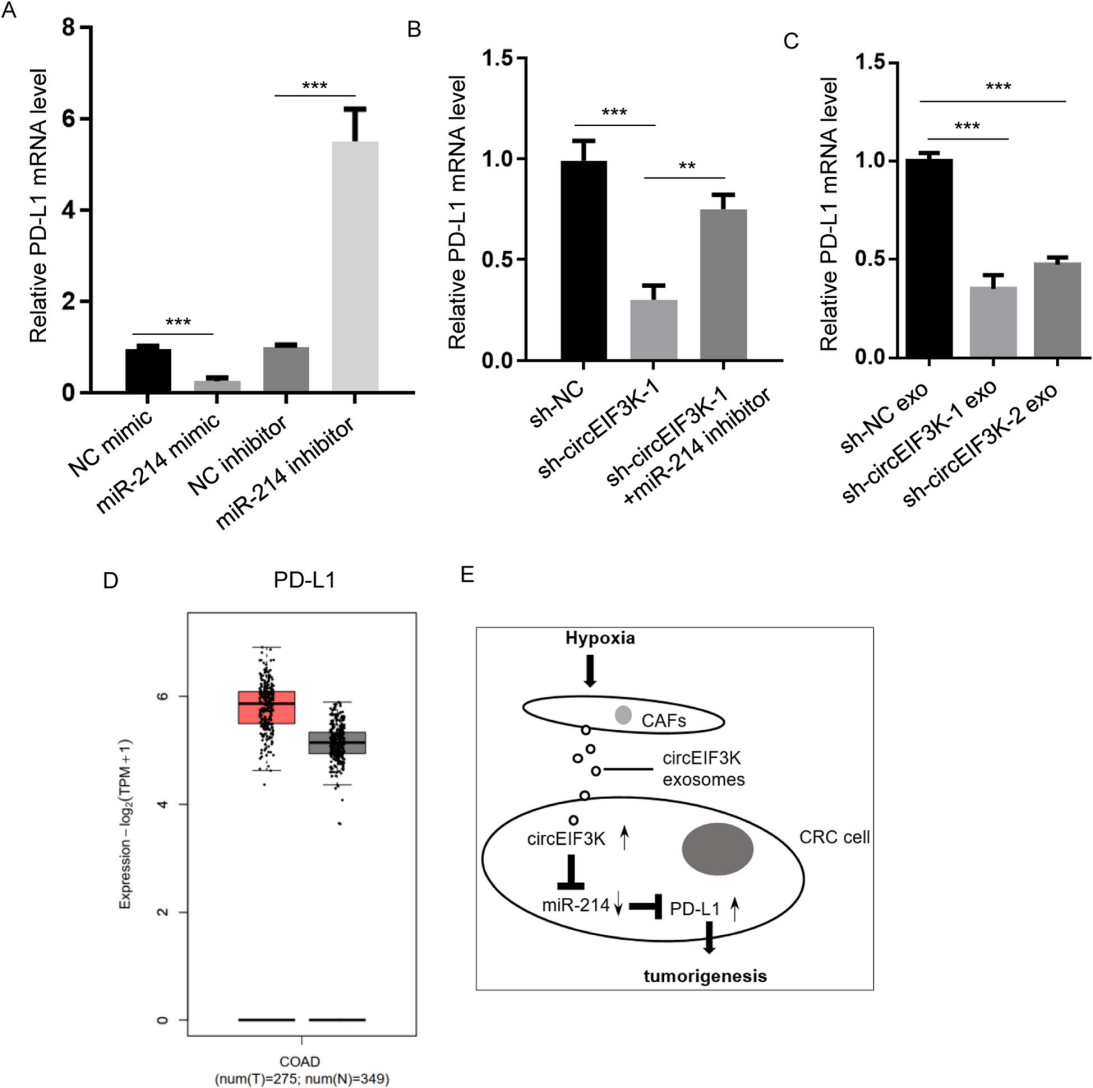
miR-214 downregulated PD-L1 expression in CRC. **A** PD-L1 expression levels were detected by RT-qPCR in HCT116 cells transiently transfected with NC mimic, miR-214 mimic, NC inhibitor, miR-214 inhibitor. ***p < 0.001. **B** PD-L1 mRNA levels were detected by RT-qPCR in HCT116 cells expressing sh-NC, sh-circEIF3K-1, sh-circEIF3K-1 plus miR-214 inhibitor. **p < 0.01; ***p < 0.001. **C** PD-L1 mRNA levels were detected by RT-qPCR in HCT116 cells incubated with exosomes derived from CAFs stably transfected with sh-NC, sh-circEIF3K-1, sh-circEIF3K-2. ***p < 0.001. **D** TCGA dataset analysis showed PD-L1 levels of CRC tumors (*n* = 275) and adjacent normal tissues (*n* = 349). **E** Working model of hypoxia-induced tumorigenesis of CRC by promoting circEIF3K exosomes secretion from CAFs

Next, we asked how miR-214 inhibited CRC progression. Previous study showed that PD-L1 was regulated by miR-214 [18]. PD-L1 level was reduced in miR-214 mimic-transfected HCT116 cells, while its level was increased by miR-214 inhibitor (Fig. 5A). In addition, we employed RT-qPCR to determine PD-L1 mRNA levels in HCT116 cells transfected with sh-NC, sh-circEIF3K or sh-circEIF3K plus miR-214 inhibitor. The result displayed decreased PD-L1 expression and partial rescue of the attenuated PD-L1 expression (Fig. 5B). Then, RT-qPCR showed lower PD-L1 levels in HCT116 cells incubated with exosomes from sh-circEIF3K CAFs compared to sh-NC (Fig. 5C). In patients, TCGA dataset showed higher level of PD-L1 in CRC tumors compared to normal tissues (Fig. 5D). To sum up, these data indicated that PD-L1 might be an potential target for circEIF3K/miR-214 axis in CRC.

## Discussion

Cancer-associated fibroblast was one of the stromal cells in tumor microenvironment that regulated proliferation, migration and metastasis of cancer cells [20]. During this process, some risk factors (e.g., hypoxia) might be important for CAFs-affected solid tumors [21]. For instances, CAFs were found to be implicated in breast cancer [22], pancreatic cancer [23], and gastric cancer [24]. However, few reports revealed the detailed and clear mechanism for explaining the role of hypoxia in promoting cancer progression via CAFs. In our study, we utilized a series of experiments to validate that hypoxia-treated CAFs could induce secretion of exosomal circEIF3K.

Exosomes are defined as secreted extracellular vesicles with 30–150 nm in diameter [25]. In which, some molecules (like DNAs, RNAs, proteins or lipids) were existed in exosomes with lipid bilayer [26]. The exosomes could transfer the molecules to surrounding cells and impact cell biology [27, 28]. We found that exosome secreted by hypoxia contained circEIF3K and transferred to CRC cells. Our findings were consistent with previous studies.

To date, circRNAs were involved in multiple biological processes. CircMTO1 regulated Wnt signaling to affect proliferation and invasion of CRC [29]. CircITGA7 suppressed CRC metastasis via regulating Ras pathway [30]. Zhang et al. showed circSMAD2 promoted CRC cells proliferation [31]. Besides, we also demonstrated that circEIF3K played a role in CRC proliferation, invasion and metastasis, which had never been reported before.

MiR-214 has been shown to inhibit cancer cell phenotypes through interacting with lncRNAs, circRNAs or others [32, 33]. In this study, miR-214 was confirmed to bind circEIF3K and could rescue circEIF3K-mediated CRC progression, which was in agreement with prior findings. On the other hand, Sun et al. showed that miR-214 targeted PD-L1 and downregulated its expression [18]. We finally confirmed this conclusion in CRC cells, which was first in CRC.

## Conclusions

In conclusion, we originally propose that circEIF3K is implicated in CRC by regulating miR-214/PD-L1 (Fig. 5E). These findings, for the first time, show the relationship between circEIF3K and CRC. At molecular level, we also creatively link circEIF3K to miR-214 and PD-L1. Thus, our research will deepen the understanding of CRC pathogenesis and help develop novel therapeutic approaches.

## Data Availability

All data generated or analysed during this study were included in this article. TCGA dataset (https://tcga-data.nci.nih.gov) was used in the study.

## Abbreviations

CRC: Colorectal cancer
CAF: Cancer-associated fibroblast
RT-qPCR: Reverse-transcription quantitative PCR
GFR: Growth Factor Reduced
TCGA: The Cancer Genome Atlas
PD-L1: Programmed death ligand-1
HDLEC: Human dermal lymphatic endothelial cells
